# Could biorational insecticides be used in the management of aflatoxigenic *Aspergillus parasiticus* and its insect vectors in stored wheat?

**DOI:** 10.7717/peerj.1665

**Published:** 2016-02-22

**Authors:** Tiyyabah Khan, Ahmad Ali Shahid, Hafiz Azhar Ali Khan

**Affiliations:** Faculty of Life Sciences, Institute of Agricultural Sciences, University of the Punjab, Lahore, Pakistan

**Keywords:** Spinosad, Indoxacarb, Thiamethoxam, Imidacloprid, Stored insect pests, Aflatoxin, Mycotoxin, *Tribolium*, *Rhyzopertha*

## Abstract

Insect pests in stored wheat cause significant losses and play an important role in the dispersal of viable fungal spores of various species including aflatoxin producing *Aspergillus parasiticus*. The problem of insecticide resistance in stored insects and environmental hazards associated with fumigants and conventional grain protectants underscore the need to explore reduced risk insecticides to control stored insects with the ultimate effect on aflatoxin production. The purpose of this study was to investigate the insecticidal potential of four biorational insecticides: spinosad, thiamethoxam, imidacloprid and indoxacarb, on wheat grains artificially infested with *Rhyzopertha dominica*/*Sitophilus oryzae* and/or *A. parasiticus* spores, and the subsequent effect on aflatoxin production. Spinosad and thiamethoxam were the most effective insecticides against *R. dominica* compared to *S. oryzae* followed by imidacloprid. Spinosad applied at 0.25–1 ppm and thiamethoxam at 2 and 4 ppm concentrations resulted in complete mortality of *R. dominica*. However, indoxacarb was more toxic against *S. oryzae* compared to *R. dominica*. Wheat grains inoculated with *R. dominica*/*S. oryzae* +spores elicited higher aflatoxin levels than wheat grains inoculated with or without insecticide+spores. In all the treatment combinations containing insects, aflatoxin production was dependent on insects’ survival rate. In addition, thiamethoxam and imidacloprid had also a significant direct effect on reducing aflatoxin production. Aflatoxin levels were lower in the treatment combinations with any concentration of thiamethoxam/imidacloprid+spores as compared to wheat grains inoculated with spores only. Correlation analyses revealed highly significant and positive association between moisture contents/insect survival rate and production of aflatoxin levels, and insect survival rate and moisture contents of the wheat grains. In conclusion, the results of the present study provide baseline data on the use of biorational insecticides against *R. dominica* and *S. oryzae* and subsequent effect on aflatoxin production.

## Introduction

Aflatoxins are the group of structurally diverse mycotoxins that are mainly produced by *Aspergillus flavus* and *A. parasiticus*, both belonging to section *Flavi* (Bennett & Klich, 2003; Horn, 2007). These mycotoxins are recognized as immunosuppressive, carcinogenic, hepatotoxic, mutagenic and teratogenic (Patten, 1981), since they lead to serious human and animal health hazards, including acute and chronic liver diseases, tumor induction, reproductive disorders, genotoxicity and nephrotoxicity (Chao, Maxwell & Wong, 1991; Lacey, 1988; Desjardins et al., 2000). These mycotoxins are also known to contaminate more than 25% of the world stored grain cereal commodities of which more than 300 fungal metabolites have been reported to cause human and animal toxicity (Galvano et al., 2001).

Stored grain contaminations with insect pests and fungi are a serious problem resulting in more than 20% losses in overall production by decreasing seed germination and downgrading of grains (Philip & Throne, 2010). Factors like the temperature and humidity at postharvest stages play an important role in insect pest infestations, growth of toxigenic fungi and aflatoxin production in storage ecosystems. Contamination of stored grains with fungal spores is mainly a source of mycotoxins which usually results from stored insect pest infestations (Birck, Lorini & Scussel, 2006). Insects disseminate the fungal spores all over the grain bulk by their constant movement, which are carried on their body and/or deposited in insect frass (Santos & Mantovani, 1997). These insects break the seed coat as a natural barrier to fungus and provide an entry point for fungal infection (Setamau et al., 1998) which ultimately results in mycotoxin production. Therefore, strategies to control insects are needed. The most serious insect pests of stored grains that cause >20% postharvest losses in developing countries are: rice weevil (*Sitophilus oryzae*), red flour beetle (*Tribolium castaneum*), lesser grain borer (*Rhyzopertha dominica*), rusty grain beetle (*Cryptolestes ferrugineus*) and khapra beetle (*Trogoderma granarium*) (Philip & Throne, 2010).

Control of these insects is of prime importance as they have the ability to carry and transmit spores of aflatoxigenic *Aspergillus* spp. internally (Barra, Nesci & Etcheverry, 2013). Current control measures for these insects rely on the extensive use of fumigants and conventional insecticides that have created some drawbacks like increased insect resistance (Gurusubramanian et al., 2008), primary pest resurgence (Hazarika, Bhuyan & Hararika, 2009), secondary pest outbreak (Cranham, 1996), and their use as grain protectants is being reconsidered for their effect on health and environmental safety (Vayias et al., 2010). Some of the conventional insecticides have also been reported to inhibit the production of aflatoxins in storage ecosystems. Dichlorvos (an organophosphate) was the first conventional insecticide reported to be effective in the inhibition of aflatoxin production in different stored commodities like corn, rice, wheat and peanuts (Rao & Hareim, 1972). Thereafter, insecticides from different classes like naled, pyrethrum, sevin, malathion, diazinon (Draughon & Ayres, 1981) and carbaryl (D’Mello et al., 1998) were reported to significantly inhibit the production of aflatoxins. However, recent reports on drawbacks (see above) of conventional insecticides raised questions about their use in the future. Therefore, there is a need to explore reduced risk or biorational insecticides with minimal environmental effect and mammalian toxicity for judicious insect pest and aflatoxin management.

Biorational insecticides are usually target specific new insecticides with low mammalian toxicity. Some of the biorational insecticides like spinosad, thiamethoxam have been proved as potential grain protectants against stored insect pests in different parts of the world (Vayias et al., 2010; Arthur, Yue & Wilde, 2004). However, such studies are rare at the Pakistan level. Therefore, in the present study, our aims were to evaluate the toxicity of spinosad, thiamethoxam, imidacloprid and indoxacarb against two major pests of stored wheat *R. dominica* and *S. oryzae*, and the subsequent effect on aflatoxin production in wheat grains. The results would be helpful in the management of insects and aflatoxins in stored commodities.

## Materials and Methods

### Commodities, formulations and fungal cultures

Clean and infestation free wheat grains (var. Seher-06) were used in the present study. The grains were surface sterilized with a 0.5% sodium hypochlorite solution for 2 min and washed with sterile distilled water before starting the experiments. The insecticide formulations were spinosad (Tracer^®^ 24% active ingredients [IA]; Arysta Life Sciences, Karachi, Pakistan), thiamethoxam (Actara^®^ 25% [AI]; Syngenta, Pakistan), imidacloprid (Confidor^®^ 20% [AI]; Bayer Crop Sciences, Karachi, Pakistan) and indoxacarb (Steward 15%, DuPont, Pakistan). The *Aspergillus parasiticus* culture was obtained from the First Fungus Culture Bank, Pakistan (FFCB), and used in bioassays.

### Insects

Healthy cultures of *R. dominica* and *S. oryzae* adults were collected from grain market, Lahore (31.5497°N, 74.3436°E), and reared on whole wheat in the laboratory at 26 ± 1 °C and 70 ± 5%. Adults of both species used in bioassays were mixed sex and 2-3-weeks-old (Athanassiou et al., 2011).

### Grain treatment and Bioassays

Insecticidal bioassays were done by following the methodology of Athanassiou et al. (2011) with some modifications. Briefly, insecticides were applied as a solution diluted with distilled water. Individual replicate lots of 200 g of wheat grains were placed in 0.5 liter glass jars for different treatment combinations (Table 1). To achieve the desired insecticide concentrations, 2 mL solution of a respective insecticide was prepared and applied to the 200 g grains. In order to maximize insecticide distribution, the jar was shaken manually for 5 min (Athanassiou et al., 2011; Krishnamurthy, Shashikala & Naik, 2008). The treatment combinations 5 and 6 (without any insecticide) were treated with distilled water. The jars were left in the laboratory for 24 h at 25 °C and complete darkness to dry. After 24 h, the jars with and without insecticide treated wheat grains were inoculated with 0.5 ml spore suspension (10^4^ spores/ml) of *A. parasiticus*, except the treatment combination 6 (i.e., wheat only). Twenty adults of *R. dominica* (arena “A”) or *S. oryzae* (arena “B”) were introduced in each jar. The insects were disinfected with 0.5% sodium hypochlorite for 2 min and washed with sterile distilled water before entering into the jars (Ferreira-Castro et al., 2012). To check the direct effect of insecticides on aflatoxin production, the same procedure was repeated, but without introducing insects in treated jars (treatment combination 7–9). The treated jars were closed with muslin cloth and maintained in the laboratory at 26 ± 1 °C and 70 ± 5%. The entire procedure was replicated five times by preparing new lots of treated wheat for each replicate of the respective treatment combination. After 14 days, the jars were opened for the determination of insects’ mortality, aflatoxin levels and moisture contents. The moisture contents were determined by following the procedure of AACC (2000) method No. 44-15.

**Table 1:** Treatment combinations used in different experiments.

Insecticide	Treatment	Combination
Spinosad	1	0.25 ppm + *R. dominica* OR *S. oryzae* + spores
	2	0.5 ppm + *R. dominica* OR *S. oryzae* + spores
	3	1 ppm + *R. dominica* OR *S. oryzae* + spores
	4	*R. dominica* OR *S. oryzae* + spores
	5	Spores only
	6	Wheat only
	7	0.25 ppm + spores
	8	0.5 ppm + spores
	9	1 ppm + spores
Imidacloprid, Thiamethoxam or Indoxacarb	1	1 ppm + *R. dominica* OR *S. oryzae* + spores
	2	2 ppm + *R. dominica* OR *S. oryzae* + spores
	3	4 ppm + *R. dominica* OR *S. oryzae* + spores
	4	*R. dominica* OR *S. oryzae* + spores
	5	Spores only
	6	Wheat only
	7	1 ppm + spores
	8	2 ppm + spores
	9	4 ppm + spores

Aflatoxin content was determined by following the methodology described by Beti, Phillips & Smalley (1995), using an indirect competitive enzyme linked immunosorbent assay (ELISA) method.

### Data analysis

Percent mortality and aflatoxin contents in different treatments were analyzed using a one-way analysis of variance (ANOVA) with Statistix 8.1 software (Analytical Software, 2005). Means were compared by Tukey’s Honestly Significant Difference (HSD) test, at 0.05 probability. Correlation analysis was used to study the relationship among aflatoxin content, grain moisture content and insects’ survival rate.

**Figure 1:**
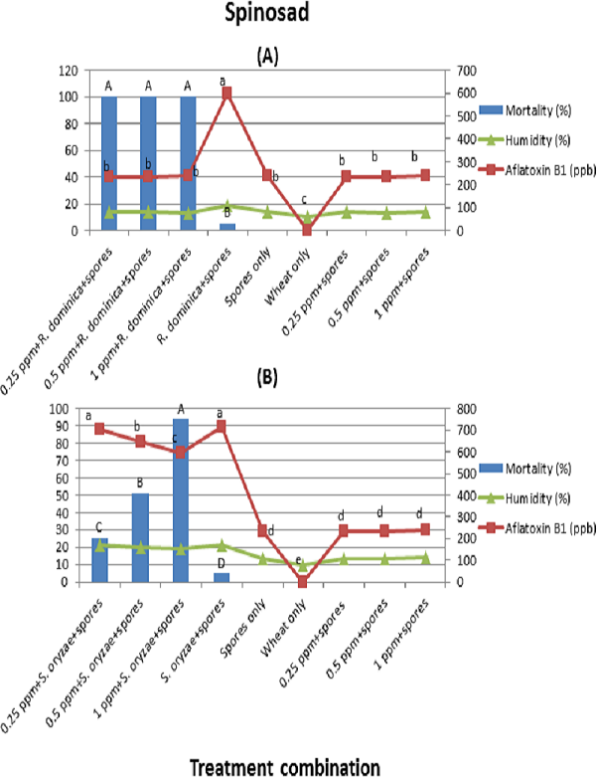
(A) and (B) (Spinosad). Effect of different treatment combinations on mortality of *R. dominica* (1A) or *S. oryzae* (1B), grain moisture contents (primary axis) and aflatoxin production levels (secondary axis). Data bars or lines with different letters are significantly different; in all 369 cases *df* = 3, 16, Tukey’s HSD test at *p* < 0.01.

## Results

### Insects’ mortality

In the case of spinosad, mortality for *R. dominica* was higher than for *S. oryzae*. *Rhyzopertha dominica* showed 100% mortality at the spinosad concentrations of 0.25–1 mg/kg (*F* = 3,610; *df* = 3, 16; *p* < 0.01) (Fig. 1A). However, *S. oryzae* showed the highest mortality at the concentration 1 ppm followed by 0.5 and 0.25 ppm (*F* = 269; *df* = 3, 16; *p* < 0.01) (Fig. 1B).

**Figure 2:**
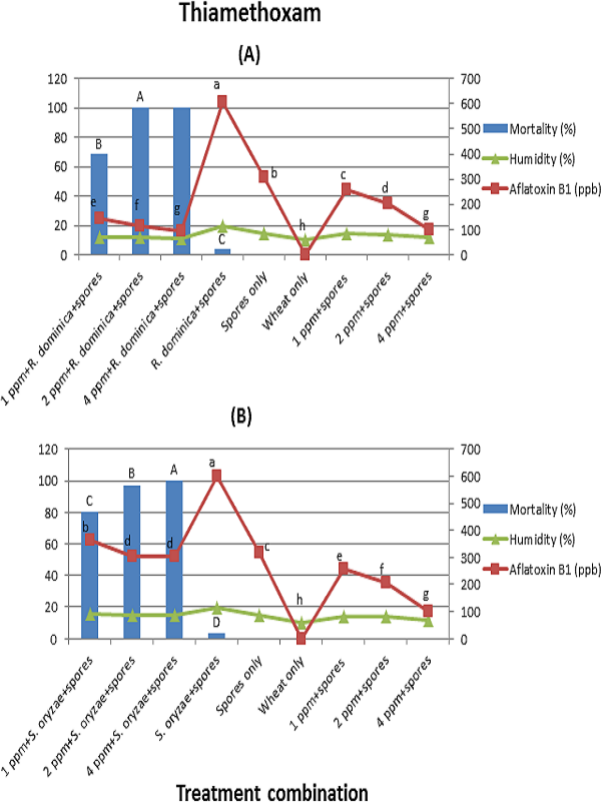
(A) and (B) (Thiamethoxam). Effect of different treatment combinations on mortality of * R. dominica* (2A) or *S. oryzae* (2B), grain moisture contents (primary axis) and aflatoxin production levels (secondary axis). Data bars or lines with different letters are significantly different; in all cases *df* = 3, 16, Tukey’s HSD test at *p* < 0.01.

*Rhyzopertha dominica* showed complete mortality on wheat treated with thiamethoxam at concentrations 2 and 4 ppm (*F* = 1,170; *df* = 3, 16; *p* < 0.01) (Fig. 2A). Whereas, *S. oryzae* showed complete mortality only at 4 ppm (*F* = 1,036; *df* = 3, 16; *p* < 0.01) (Fig. 2B).

**Figure 3:**
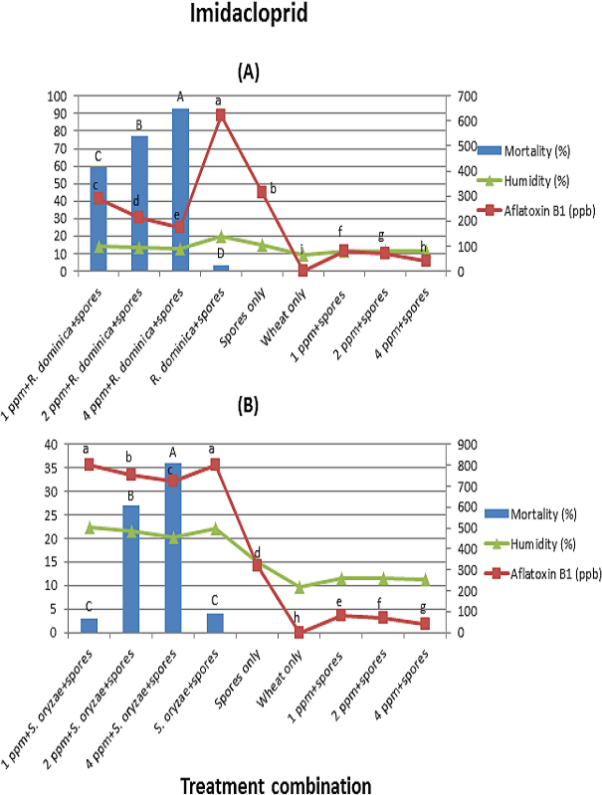
(A) and (B) (Imidacloprid). Effect of different treatment combinations on mortality of *R. dominica* (3A) or *S. oryzae* (3B), grain moisture contents (primary axis) and aflatoxin production levels (secondary axis). Data bars or lines with different letters are significantly different; in all cases *df* = 3, 16, Tukey’s HSD test at *p* < 0.01.

In the case of imidacloprid, mortality for *R. dominica* was higher than for *S. oryzae*. *R. dominica* showed 93% mortality at the highest concentration 4 ppm (*F* = 473; *df* = 3, 16; *p* < 0.01) (Fig. 3A). Whereas, *S. oryzae* showed 36% mortality at the highest concentration 4 ppm (*F* = 88.0; *df* = 3, 16; *p* < 0.01) (Fig. 3B).

**Figure 4:**
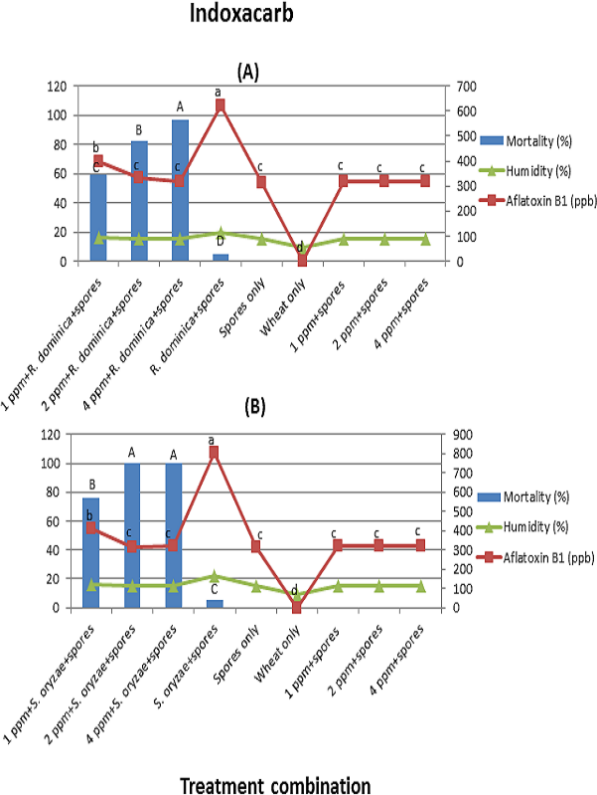
(A) and (B) (Indoxacarb). Effect of different treatment combinations on mortality of *R. dominica* (4A) or *S. oryzae* (4B), grain moisture contents (primary axis) and aflatoxin production levels (secondary axis). Data bars or lines with different letters are significantly different; in all cases *df* = 3, 16, Tukey’s HSD test at *p* < 0.01.

For indoxacarb, in contrast, mortality for *R. dominica* was lower than for *S. oryzae*. *R. dominica* showed 97% mortality at the highest concentration 4 ppm (*F* = 302; *df* = 3, 16; *p* < 0.01) (Fig. 4A). However, *S. oryzae* showed complete mortality at 2 and 4 ppm (*F* = 437; *df* = 3, 16; *p* < 0.01) (Fig. 4B).

### Aflatoxin B1 production

Wheat grains inoculated with *R. dominica*/*S. oryzae* + spores elicited higher aflatoxin levels than wheat grains inoculated with any concentration of spinosad + spores. In the case of concentrations of spinosad + spores + *R. dominica*/*S. oryzae*, aflatoxin production was dependent on insect survival rate. In the case of *R. dominica*, where 100% mortality was observed at all the concentrations of spinosad, aflatoxin production was lower as compared to the treatment combination where wheat grains were inoculated with *R. dominica* + spores. The same trend was observed in the treatment combinations where *S. oryzae* were inoculated. Spinosad concentrations seem to have no direct effect on aflatoxin production. For instance, there was no statistical difference between concentrations of spinosad + spores and wheat grains inoculated with spores only in both arenas. The average moisture contents in the wheat grain were ranged between 10–19% and 9.5–21% in arena A and B, respectively. The moisture contents were higher where survival percentage of insects was high compared to those treatments where the survival percentage was low or no insects inoculated (Figs. 1A and 1B).

In the case of thiamethoxam and imidacloprid, wheat grains inoculated with *R. dominica*/*S. oryzae* + spores elicited higher aflatoxin levels than the rest of the treatment combinations. In the case of treatment combination containing insects in both arenas, aflatoxin levels were dependent on insect survival rate. However, thiamethoxam and imidacloprid concentrations had also a significant direct effect on reducing aflatoxin production. Aflatoxin levels in both arenas were lower in the treatment combinations with any concentration of thiamethoxam/imidacloprid + spores as compared to wheat grains inoculated with spores only. Moreover, aflatoxin levels were reduced with increasing concentrations of both insecticides. The average moisture contents in the wheat grain were ranged between 9.5–20.1% and 9.7–22.25% in arena A and B, respectively.

Similar to previous experiments, wheat grains inoculated with *R*. dominica/S. *oryzae* + spores elicited higher aflatoxin levels than the rest of the treatment combinations, and the levels were dependent on insects’ survival rate. However, in contrast to thiamethoxam and imidacloprid, indoxacarb did not have a significant direct effect on reducing aflatoxin levels when compared to the treatments where wheat grains were inoculated with spores only. The average moisture contents of wheat grains were ranged between 9.5–19.7% and 9.5–22.3% in arena A and B, respectively (Figs. 4A and 4B).

### Correlation analyses

Correlation analyses revealed highly significant and positive association between moisture contents/insect survival rate and production of aflatoxin levels, and insect survival rate and moisture contents of the wheat grains (Table 2).

**Table 2:** Correlation analyses among insects’ mortality, grain moisture contents and aflatoxin production in different treatment combinations.

	Aflatoxin	Moisture content
Moisture content	0.99^**^	–
*Ryzopertha dominica*	0.91^**^	0.92^**^
*Sitophilus oryzae*	0.84^**^	0.86^**^

**Notes.**

** *p* < 0.01.

## Discussion

The results of the present study revealed varying level of toxicity of insecticides against * R. dominica* and *S. oryzae*. The results also suggest that control of these insects may help limit the production of aflatoxins in stored wheat.

Overall, spinosad was the most effective insecticide against *R. dominica* compared to *S. oryzae*. Spinosad applied at 0.25, 0.5 and 1 ppm concentrations resulted in complete mortality of *R. dominica*. In contrast, *S. oryzae* showed 94% mortality at the highest concentration of spinosad tested. Spinosad is a relatively new insecticide of low mammalian toxicity and has a broad spectrum of target species, including stored insect pests (Athanassiou, Kavallieratos & Chintzoglou, 2008), and has been registered for use against stored insect pests in the United States (Athanassiou et al., 2011). The findings of spinosad toxicity against tested insect species are in agreement with a number of previous reports (Athanassiou et al., 2011; Athanassiou, Kavallieratos & Chintzoglou, 2008; Athanassiou et al., 2008; Fang, Subramanyam & Arthur, 2002; Nayak, Daglish & Byrne, 2005). It seems that, if *R. dominica* is the sole species in stored commodities, spinosad may be as an effective grain protectant.

In the case of thiamethoxam, *R. dominica* showed complete mortality at concentrations 2 and 4 ppm, whereas *S. oryzae* showed complete mortality at only 4 ppm. Thiamethoxam is a neonicotinoid and a relatively new insecticide of low mammalian toxicity and does not produce mutagenic and/or teratogenic effects (Arthur, Yue & Wilde, 2004). It has been extensively used as a seed coat treatment with satisfactory results against a wide range of pests (Khan et al., 2015). Thiamethoxam was the first time evaluated as a grain protectant by Arthur, Yue & Wilde (2004) and found very effective against *R. dominica* and *S. oryzae*. It was further reported that the mortality of *S. oryzae* and *R. dominica* increased with time and reached approximately 100% after 6 d of exposure to thiamethoxam. But in our study, *S. oryzae*, showed less than 100% mortality at 2 ppm after 14 d of exposure. This difference could be due to the different origin of *S. oryzae* strains.

Imidacloprid is also a neonicotinoid with low mammalian toxicity and has been used as a seed treatment for the management of a number of sucking insect pests (Khan et al., 2015). Recently, Daglish & Nayak (2012) investigated the potential of imidacloprid against different beetle pests of stored grains. They reported that the toxicity of imidacloprid was species and dose dependent, and that *R. dominica* was more susceptible to imidacloprid than *S. oryzae*. The results of the present study with respect to imidacloprid also revealed that the mortality for *R. dominica* was higher than for *S. oryzae*. *Rhyzopertha dominica* showed up to 94% mortality at the highest concentration 4 ppm as compared to 39% mortality for *S. oryzae*.

Indoxacarb belongs to an oxadiazine insecticide group and has strong potential against different insect pests (Khan, Shad & Akram, 2013). In the present study mortality for * R. dominica* was lower than for *S. oryzae*, which is in agreement with Daglish & Nayak (2012) who evaluated indoxacarb potential as a grain protectant first time.

Concerning the aflatoxin production in different treatment combinations, production of aflatoxin was directly related to survival rate of both insect species. Moreover, a highly significant and positive association was observed between moisture contents/insect survival rate and production of aflatoxin levels, and insect survival rate and moisture contents of wheat grains. There was an indirect effect of spinosad and indoxacarb on the inhibition aflatoxin production. These insecticides caused mortality of the insect species which might result in the reduction of aflatoxin production. One most probable reason could be that increased mortality of insects resulted in the reduced insects’ movement, humidity and seed coat damage which ultimately affect fungal infection and aflatoxin production. However, thiamthoxam and imidacloprid also showed a direct effect on the inhibition of aflatoxin production in the treated grains. After the harvest of the crops, temperature and humidity play an important role in the growth of toxigenic fungi, aflatoxin production and insects in the storage ecosystem (Nesci, Barra & Etcheverry, 2011). Stored insect pests in the granary ecosystem move for breeding and feeding purposes from one point another and contribute the dispersal of viable spores of fungi of various species, including *A. parasiticus*, which are carried on the insect body surface or deposited in their frass (Nesci, Barra & Etcheverry, 2011). During feeding in stored commodities, insects break the seed coat of grains, which is a natural barrier to fungal growth, and ultimately promote the easy spread of mycotoxin producing fungal spores (Setamau et al., 1998). Hence, in the present study aflatoxin production was more in those treatments where the survival rate of insects was high, most probably due to a high rate of feeding and movement activities. Dispersal of fungal spores usually depends on the type of insect species present in storage commodities (Lamboni & Hell, 2009). During storage, the invasion by a variety of insects causes losses to storage grains and act as mechanical vectors of viable fungal spores (Ferreira-Castro et al., 2012). If storage grains left untreated, insects within the granary ecosystem can create local conditions of humidity and temperature that favor the rapid growth of fungi, resulting in deterioration of the grain mass and production of mycotoxins (Sinha & Sinha, 1992). The results of the correlation in the present study are in accordance with those of Beti et al. (Daglish & Nayak, 2012) who reported a highly significant and positive association between moisture contents/insect survival rate and production of aflatoxin levels, and insect survival rate and moisture contents of the wheat grains.

Since the insecticides tested in the present study are not recommended yet as grain protectants in Pakistan, further studies should be conducted to assess their long term efficacy or persistence against different stored pests across different stored grain commodities. Spinosad and thiamethoxam proved very effective against *R. dominica*. In granary systems where *R. dominica* is the sole problem, spinosad and thiamethoxam may provide adequate protection. However, tested insecticides except indoxacarb did not provide adequate control of *S. oryzae*. Therefore, in storage bins where both insect species coexist, there is a need to evaluate other insecticides or insecticide combinations which can provide adequate protection against insect pests and subsequent fungal infection. In conclusion, keeping in view the role of stored insects in aflatoxin production, the results of the present study provide baseline data on the use of biorational insecticides against *R. dominica* and *S. oryzae* and the subsequent effect on the inhibition of aflatoxin production. Whether this is a practical solution in granary systems for a long time needs to be further characterized in future studies.

## Supplemental Information

10.7717/peerj.1665/supp-1 Data S1 Raw data

## Additional Information and Declarations

### Data Availability

The following information was supplied regarding data availability: Raw data has been provided in the Supplemental Information.
