# Supplementary material for: Could biorational insecticides be used in the management of aflatoxigenic Aspergillus parasiticus and its insect vectors in stored wheat?
Source: PeerJ. 2016 Feb 22;4:e1665. doi: 10.7717/peerj.1665 (PMC4768669; doi:10.7717/peerj.1665)
Supplement: Data S1 [file peerj-04-1665-s001.pdf]

	**spinosad**						
		r.dominica			s oryz		
trt	repl	mort	permort	afla	mort	permort1	afla
1	1	20	100	236	5	25	703
1	2	20	100	239	4	20	715
1	3	20	100	231	5	25	699
1	4	20	100	240	6	30	710
1	5	20	100	229	5	25	707
2	1	20	100	242	11	55	651
2	2	20	100	235	10	50	655
2	3	20	100	234	12	60	645
2	4	20	100	240	8	40	646
2	5	20	100	231	10	50	650
3	1	20	100	240	18	90	590
3	2	20	100	229	20	100	598
3	3	20	100	233	20	100	595
3	4	20	100	243	18	90	595
3	5	20	100	244	18	90	591
4	1	1	5	601	0	0	711
4	2	1	5	590	1	5	720
4	3	2	10	585	1	5	710
4	4	0	0	605	2	10	717
4	5	1	5	600	1	5	722
5	1			242			239
5	2			240			229
5	3			236			235
5	4			229			242
5	5			242			239
6	1			0			0
6	2			0			0
6	3			0			0
6	4			0			0
6	5			0			0
7	1			229			229
7	2			235			235
7	3			234			234
7	4			240			240
7	5			240			240
8	1			236			236
8	2			239			239
8	3			229			229
8	4			235			235
8	5			234			234
9	1			240			240
9	2			229			229
9	3			242			242
9	4			245			245
9	5			240			240

imidacoprid

trt	repl	r.dominica mort	permort	afla	s oryz mort	permort	afla
1	1	10	50	292	0	0	795
1	2	12	60	285	1	5	807
1	3	12	60	282	1	5	802
1	4	13	65	286	0	0	799
1	5	12	60	286	1	5	805
2	1	15	75	211	6	30	760
2	2	16	80	209	6	30	751
2	3	16	80	215	6	30	757
2	4	14	70	212	5	25	755
2	5	16	80	216	4	20	752
3	1	18	90	173	7	35	724
3	2	19	95	169	8	40	720
3	3	19	95	175	7	35	725
3	4	18	90	173	8	40	724
3	5	19	95	178	6	30	725
4	1	0	0	622	0	0	800
4	2	1	5	628	2	10	803
4	3	1	5	620	1	5	791
4	4	1	5	625	1	5	797
4	5	0	0	620	0	0	801
5	1			325			318
5	2			318			315
5	3			316			317
5	4			312			314
5	5			307			322
6	1			0			0
6	2			0			0
6	3			0			0
6	4			0			0
6	5			0			0
7	1			81			81
7	2			85			85
7	3			78			78
7	4			78			78
7	5			80			80
8	1			75			75
8	2			70			70
8	3			68			68
8	4			71			71
8	5			71			71
9	1			38			38
9	2			44			44
9	3			41			41
9	4			42			42
9	5			39			39

Thiamethoxam

		r.dominica			s oryz			
trt	repl	mort	permort	afla	mort	permort	afla	
1	1	14	70	140	15	75	363	
1	2	14	70	145	16	80	370	
1	3	13	65	150	17	85	359	
1	4	15	75	142	16	80	366	
1	5	13	65	151	16	80	364	
2	1	20	100	111	18	90	298	
2	2	20	100	113	19	95	310	
2	3	20	100	120	20	100	311	
2	4	20	100	115	20	100	304	
2	5	20	100	115	20	100	306	
3	1	20	100	99	20	100	300	
3	2	20	100	103	20	100	295	
3	3	20	100	95	20	100	304	
3	4	20	100	91	20	100	306	
3	5	20	100	94	20	100	308	
4	1	1	5	610	1	5	593	
4	2	0	0	614	1	5	605	
4	3	1	5	607	1	5	600	
4	4	2	10	608	0	0	602	
4	5	0	0	605	0	0	604	
5	1			308			320	
5	2			305			320	
5	3			315			311	
5	4			310			314	
5	5			315			314	
6	1			0			0	
6	2			0			0	
6	3			0			0	
6	4			0			0	
6	5			0			0	
7	1			250			250	
7	2			260			260	
7	3			255			255	
7	4			262			262	
7	5			261			261	
8	1			201			201	
8	2			199			199	
8	3			208			208	
8	4			210			210	
8	5			213			213	
9	1			92			92	
9	2			100			100	
9	3			97			97	
9	4			101			101	
9	5			98			98	

indoxacarb

trt	repl	r.dominica mort	permort	afla	s oryz mort	permort	afla
1	1	10	50	401	15	75	402
1	2	11	55	406	18	90	410
1	3	13	65	395	13	65	415
1	4	13	65	390	15	75	409
1	5	12	60	399	15	75	408
2	1	16	80	252	20	100	325
2	2	16	80	345	20	100	318
2	3	17	85	350	20	100	314
2	4	15	75	358	20	100	320
2	5	18	90	350	20	100	312
3	1	20	100	317	20	100	314
3	2	19	95	322	20	100	319
3	3	20	100	325	20	100	320
3	4	18	90	318	20	100	320
3	5	20	100	314	20	100	325
4	1	0	0	631	0	0	799
4	2	1	5	622	1	5	799
4	3	1	5	625	2	10	805
4	4	1	5	618	1	5	812
4	5	2	10	620	1	5	800
5	1			309			313
5	2			306			315
5	3			309			307
5	4			316			316
5	5			317			316
6	1			0			0
6	2			0			0
6	3			0			0
6	4			0			0
6	5			0			0
7	1			315			315
7	2			321			321
7	3			317			317
7	4			322			322
7	5			319			319
8	1			325			325
8	2			318			318
8	3			314			314
8	4			319			319
8	5			320			320
9	1			322			322
9	2			324			324
9	3			317			317
9	4			320			320
9	5			319			319
